# Editorial: Climate impact on plant holobiont: Mitigation strategies and sustainability

**DOI:** 10.3389/fmicb.2022.1040876

**Published:** 2023-01-23

**Authors:** D. K. Choudhary, Anukool Vaishnav, Shekhar Jain, Mihir K. Mandal, Ram Prasad

**Affiliations:** ^1^Amity Institute of Microbial Technology, Amity University, Noida, India; ^2^Department of Biotechnology, GLA University, Mathura, Uttar Pradesh, India; ^3^Faculty of Life Sciences, Mandsaur University, Mandsaur, India; ^4^Crop Improvement and Protection Research, Agricultural Research Service (USDA), Salinas, CA, United States; ^5^Mahatma Gandhi Central University, Motihari, Bihar, India

**Keywords:** soil microbiome, plant holobiont, climate changes, agricultural sustainability, food security

Currently, the food security goal is responsible for an advanced agricultural transformation, wherein climate activity and its impact on plant and soil microbiomes are the main points of focus. Studies on plant-soil-microbe interaction provide an opportunity to study climate change mitigation strategies in new ways. Researchers around the globe are collectively thinking of how to implement holistic mitigation strategies and, henceforth, the development of sustainable agroecosystems under the impacts of climate change. One basic question that researchers should seriously consider is how the existing impacts of climate change affect the plant holobiont at the agroecosystem level. Researchers must take an interest in skill-based approaches in order to promote and enhance the plant holobiont relationship for agricultural benefits. A diverse range of microbes within the plant holobiont and their engagement under habitat-imposed stresses are beneficial to the agricultural sustainability. Furthermore, the coordination of agro-policies, procedures, and activities that encourage the microbiome application in the plant system under climate change is necessary.

With the above information in mind, the present editorial is designed to discuss the effective, cognitive, and scientific progression of the impact of climate change on the plant holobiont. Through the deployment of activity- and skill-based approaches to structural and functional microbial attributes, we may be able to develop strategies for ensuring the sustainability of agroecosystems. To facilitate our understanding of the impact of climate change on the plant holobiont, we hereby present a collection of basic, applied research, which we hope will ignite a scientific discussion. Here, we argue that knowledge of habitat-imposed stresses and their mitigation strategies is indispensable to the sustainability of agriculture and that there is a need for scientific input in the form of basic and applied research in order to better understand the situation surrounding food security under the impacts of climate change.

We invited manuscripts based on this theme to uncover the structural and functional attributes of soil and plant microbiomes and their reactions when subjected to climate change. It is our belief that this kind of compendium is required in order to achieve sustainable development goals (e.g., 1, 2, 11, 13, and 15), which foster the transfer of knowledge between scientific communities, industries, and young researchers and students working in this field.

The research and review papers included in the present Research Topic were compiled with the following objectives in mind:

- Discuss the impact of habitat-imposed stresses on relationships within the plant holobiont.
- Discuss the sustainability of agroecosystem under climate change.
- Explore the richness of soil microbiomes and its impact on soil and plant productivity.
- Elaborate on the ecophysiology of the soil microbiome under various climatic changes, i.e., habitat- imposed stress.
- Determine the below-ground impact due to climate changes in aboveground.

The following introductions to the articles selected for this Research Topic provide an insight into the topics discussed therein:

Abbas et al. contributed to this Research Topic with a paper titled “Root rot as a silent alfalfa killer in China: Distribution, fungal, and oomycete pathogens, impact of climatic factors and its management,” wherein the authors emphasize the impact of biotic stress and its mitigation. In this study, the authors describe the various impacts of climate change on alfalfa lines/cultivars with regard to resistance to a diverse range of pathogens. In addition, they highlight the alfalfa quantitative trait loci (QTL) against resistance and susceptibility to root rot pathogens.

Saud et al. compiled their research in a paper titled “Comprehensive Impacts of climate change on rice production and adaptive strategies in China,” which focuses on abiotic stress and its impact on rice cultivation in China. In this study, the authors describe a technique known as climate-smart rice construction which is used for forecasting, rice plantation, and enhancing comprehensive ability.

Sudha et al. discuss “Unraveling the tripartite interaction of volatile compounds of *Streptomyces rochei* with grain mold pathogens infecting Sorghum,” wherein they show how *Streptomyces rochei* exhibits hyperparasitism, competition, and antibiosis via microbial-volatile organic compounds (mVOCs), together with their antimicrobial functions, could also enhance plant growth.

Malviya et al. attempt to understand the mechanism(s) involved in unraveling the mechanism of sulfur nutrition in pigeon pea inoculated with sulfur-oxidizing bacteria, describing the role of *S. maltophilia* and *S. pavanii* in the alteration of the root architecture of pigeon pea to verify the efficiency of sulfur uptake.

Zhang et al. define “Nematicidal activity of *Burkholderia arboris* J211 against *Meloidogyne incognita* on Tobacco” by studying a microbial-produced plant hormone and its bionematicidal activity. The authors also describe PGP activities associated with *B. arborius*.

Srivastava et al. stringently discuss “Transcriptome analysis to understand salt stress regulation mechanism of *Chromohalobacter salexigens* ANJ207,” wherein they explore the genes incurred against salt stress. Their findings reveal an increase in the transcript of genes involved in the biosynthesis of GBC and those responsible for the uptake of OpuAC, OpuAA, and OpuAB. The increased expression of compatible solute genes in high salt concentration might be responsible for the salinity adaptation in *C. salexigens* ANJ207.

Khumairah et al. describe how “Halotolerant plant growth-promoting rhizobacteria isolated from saline soil improve nitrogen fixation and alleviate salt stress in rice plants,” with an emphasis on the isolation of halotolerant PGPRs, e.g., *P. stutzeri* and *K. pneumonia*, which produce a wide range of PGP metabolites and antioxidant enzymes to ameliorate rice crop under climate changes.

Singh et al. elaborately explain “Mechanistic Insights and Potential Use of Siderophores Producing Microbes in Rhizosphere for Mitigation of Stress in Plants Grown in Degraded Land,” highlighting bacterial iron chelator (i.e., BS) and emphasizing the biochemical and genetic regulation of BS with PS in terms of cross-talk under Fe-deficient degraded land.

Abbas et al. in their contribution highlights “*Trichoderma* spp. Genes Involved in the Biocontrol Activity Against *Rhizoctonia solani,”* wherein emphasis is placed on fungal-mediated induced systemic resistance in plants through the deployment of genes in signal transduction through G protein-coupled/cAMP receptors. They also discuss the involvement of genes in the production of extracellular enzymes as biocontrol action along with their involvement in the synthesis of polyketides and non-ribosomal peptides.

Ali et al. focus on the “Induction of Systemic Resistance in Maize and Antibiofilm Activity of Surfactin from *Bacillus velezensis* MS20,” wherein the vital role of biosurfactant (surfactin) in biocontrol action for the sustainable production of maize was highlighted.

Malviya et al., in their contribution, elaborately describe “A Comparative Analysis of Microbe-Based Technologies Developed at ICAR-NBAIM Against *Erysiphe necator* Causing Powdery Mildew Disease in Grapes (*Vitis vinifera* L.),” wherein emphasis is placed on alleviating biotic stress. In this study, the authors deployed microbe-based technologies, namely Eco-pesticide^®^, Bio-Pulse^®^, and Bio-Care 24^®^ to alleviate powdery mildew at every stage of the grapevine.

Salvi et al. discuss the role of “Advancement in the molecular perspective of plant-endophytic interaction to mitigate drought stress in plants,” wherein they focus on the deployment of endophytes to alleviate abiotic stress.

Solanki et al. define the functional role of biotic stress in the tomato plant rhizosphere by highlighting the “Functional Interplay between Antagonistic Bacteria and *Rhizoctonia solani* in the tomato plant rhizosphere.” As part of this study, a field experiment was conducted with two antagonistic bacteria (*Pseudomonas* and *Bacillus*) inoculated in healthy and *Rhizoctonia-solani*-treated soil in tomato rhizosphere in order to understand the metabolic pattern and microbial function of plant disease suppression.

Bhupenchandra et al. attempt to uncover the impact of boron (B) on soil resiliency with their paper titled “Elucidating the impact of boron fertilization on soil physico-chemical and biological entities undercauliflower-cowpea-okra cropping system in an Eastern Himalayan acidic Inceptisol”. The authors performed a field experiment to assess the direct and residual implications of graded levels of applied-B on soil biological entities and their concomitant effects on crop productivity.

Shree et al. compiled their research in a paper titled “Impact of key parameters involved with plant-microbe interaction in context to global climate change,” wherein an emphasis is placed on a systemic approach to climate adaptation, which acknowledges the multidimensional nature of plant-microbe-environment interactions under stress in the development of resistant crops/plants, both now and in the future.

Through this ambitious compilation, researchers are equipped to facilitate the governance and management of climate change in line with SDGs in agriculture. This compendium is focused on plant and soil microbiomes and their role in mitigating the effects of climate change on plants. The findings of these research papers support stakeholders to enhance cooperation among institutions. Furthermore, these findings may help strengthen the management of climate change policies for sustainability. Due to the emerging effects of climate change on agricultural productivity, plants and microbiomes are valuable resources for use in sustainable agriculture, but there are also significant challenges that require new and innovative solutions.

Finally, we would like to express our profound thanks to all contributors and reviewers for their valuable time and expertise, which make this topic presentable and interesting for readers.

## Author contributions

All authors listed have made a substantial, direct, and intellectual contribution to the work and approved it for publication.

